# Inappropriate Antibiotic Prescribing in Primary Care: A Systematic Review and Implications for Clinical Practice

**DOI:** 10.7759/cureus.108853

**Published:** 2026-05-14

**Authors:** Besan M Khanfar, Rasha Ali

**Affiliations:** 1 General Practice, Independent General Practitioner, Tabuk, SAU; 2 Family Medicine, Family Medicine Academy, Tabuk Health Cluster, Tabuk, SAU

**Keywords:** antibiotic prescription, antimicrobial resistance, antimicrobial stewardship, broad-spectrum antibiotics, inappropriate antibiotic prescribing, prescribing behavior, primary care, respiratory tract infections

## Abstract

Inappropriate antibiotic prescribing in primary care is a major global problem, as primary care represents the first point of contact between the patient and a health system, contributing to health system burden and resulting in antimicrobial resistance. The objective of this study is to systematically review evidence on inappropriate antibiotic prescribing in primary care. In accordance with the Preferred Reporting Items for Systematic Reviews and Meta-Analyses (PRISMA) 2020 recommendations, PubMed and Google Scholar were searched for relevant studies that were published in the last five years, and 12 studies that fulfilled the inclusion criteria were included. Inappropriate antibiotic prescription was highly prevalent across various income settings, particularly for respiratory tract infections such as bronchitis. Broad-spectrum antibiotics, especially amoxicillin-clavulanate, were frequently inappropriately prescribed. Factors affecting inappropriate prescribing were a combination of patient, provider, and system-level determinants. Multifaceted strategies should be considered to help improve inappropriate antibiotic prescribing in primary care.

## Introduction and background

Inappropriate antibiotic use is an umbrella term that encompasses several scenarios, including inappropriate indication (antibiotic overuse) and inappropriate choice, selection, dosing, or dispensing (antibiotic misuse), as well as the lack of patient adherence [1]. Inappropriate antibiotic use is the primary driver of antimicrobial resistance [2], particularly bacterial resistance, a phenomenon in which bacteria do not respond to treatment. When antibiotics and other antimicrobials become ineffective, infections become more difficult to treat, and it becomes increasingly complicated to perform life-saving procedures, such as cancer chemotherapy, cesarean section, and other surgeries, contributing to higher morbidity and mortality [3]. Bacterial antimicrobial resistance specifically has been estimated to contribute to 4.95 million deaths globally [4].

In addition to its role in antimicrobial resistance, inappropriate antibiotic exposure can result in a potentially life-threatening infection, *Clostridioides difficile*. Evidence suggests that a pediatric patient who received inappropriate antibiotics for pharyngitis is eight times more likely to develop a *C. difficile *infection, and an adult patient is three times more likely to develop it [5]. 

Inappropriate antibiotic prescribing also contributes to an unnecessary increase in annual healthcare costs. In 2017, inappropriate antibiotic prescribing in pediatric patients alone was estimated to cost USD 74 million in excess healthcare costs, and in adult patients, USD 69 million [5]. Furthermore, using antibiotics inappropriately or for an excessive duration of time can result in inaccurate allergy documentation. As a result, future patients may receive suboptimal antibiotic therapy, increasing the risk of treatment failures and antimicrobial-resistant infections [6].

Despite growing evidence on inappropriate antibiotic use, existing literature varies widely in definitions, settings, and outcomes, making comparisons difficult. In addition, recent evidence has not been consistently synthesized with a focus on real-world prescribing patterns in primary care. As most human antibiotic use is attributed to outpatient settings [7], this review was conducted to investigate and summarize recent evidence on the prevalence, patterns, and factors associated with inappropriate antibiotic prescribing in primary care to inform future interventions.

## Review

Materials and methods

Search Strategy

The study followed the Preferred Reporting Items for Systematic Reviews and Meta-Analyses (PRISMA) guidelines. A comprehensive search for relevant articles was conducted in PubMed and Google Scholar. On Google Scholar, the keywords "inappropriate antibiotic use" and "primary care" were used. Due to the large number of results retrieved and the limited filtering capabilities of Google Scholar, the first 50 results sorted by relevance were screened. Using predefined inclusion and exclusion criteria, the titles and abstracts were assessed, and potentially eligible studies were reviewed in full text. In PubMed, the search was conducted using both free-text terms and Boolean operators. The keywords included "inappropriate antibiotic use", "antibiotic misuse", "antibiotic overuse", and "inappropriate antibiotic prescribing", combined with "primary care", "family medicine", and "general practice". No restriction on study design was chosen to maximize sensitivity. The search was filtered to only include studies published in the English language, studies on human subjects, with full text availability, and from the last five years (2021-2025). On PubMed, the search string was ("inappropriate antibiotic use"[Title/Abstract] OR "antibiotic misuse"[Title/Abstract] OR "antibiotic overuse"[Title/Abstract] OR "inappropriate antibiotic prescribing"[Title/Abstract]) AND ("primary care"[Title/Abstract] OR "family medicine"[Title/Abstract] OR "general practice"[Title/Abstract]).

Eligibility Criteria

Inclusion criteria encompassed observational studies conducted in primary care settings or primary-equivalent outpatient settings, including family medicine, internal medicine, and general practice clinics. The population included patients of all ages, including adult, pediatric, or mixed populations. Studies that reported inappropriate, unnecessary, or non‑guideline‑concordant antibiotic prescribing were included. Data sources included observed prescribing data, such as electronic health records, prescription databases, and chart reviews. The timeframe included studies published in English and between 2021 and 2025. Studies were excluded if they were not in English or published outside the timeframe (2021-2025), conducted in an inpatient hospital setting or an outpatient specialty clinic, or interventional, randomized controlled trials, surveys, vignettes, opinion pieces, editorials, or narrative reviews. Additionally, those that did not report inappropriate, unnecessary, or non‑guideline‑concordant antibiotic prescribing or lacked a clear definition of prescribing appropriateness or relied on self‑reported prescribing intentions, hypothetical scenarios, or questionnaires rather than observed prescribing data.

Quality Assessment and Risk of Bias

The 12 included studies were assessed for methodological quality using a modified Newcastle-Ottawa Scale (NOS) adapted for observational studies. Each study was assigned a score ranging from 0 to 10, with higher scores indicating better methodological quality. All included studies scored a total score of six or higher, indicating moderate to high methodological quality. The assessment included three domains: selection of the study population, comparability of study groups, and outcome assessment. Studies were then categorized as high quality (≥ 8), moderate quality (6-7), or low quality (< 6).

Study Selection

**Figure 1:**
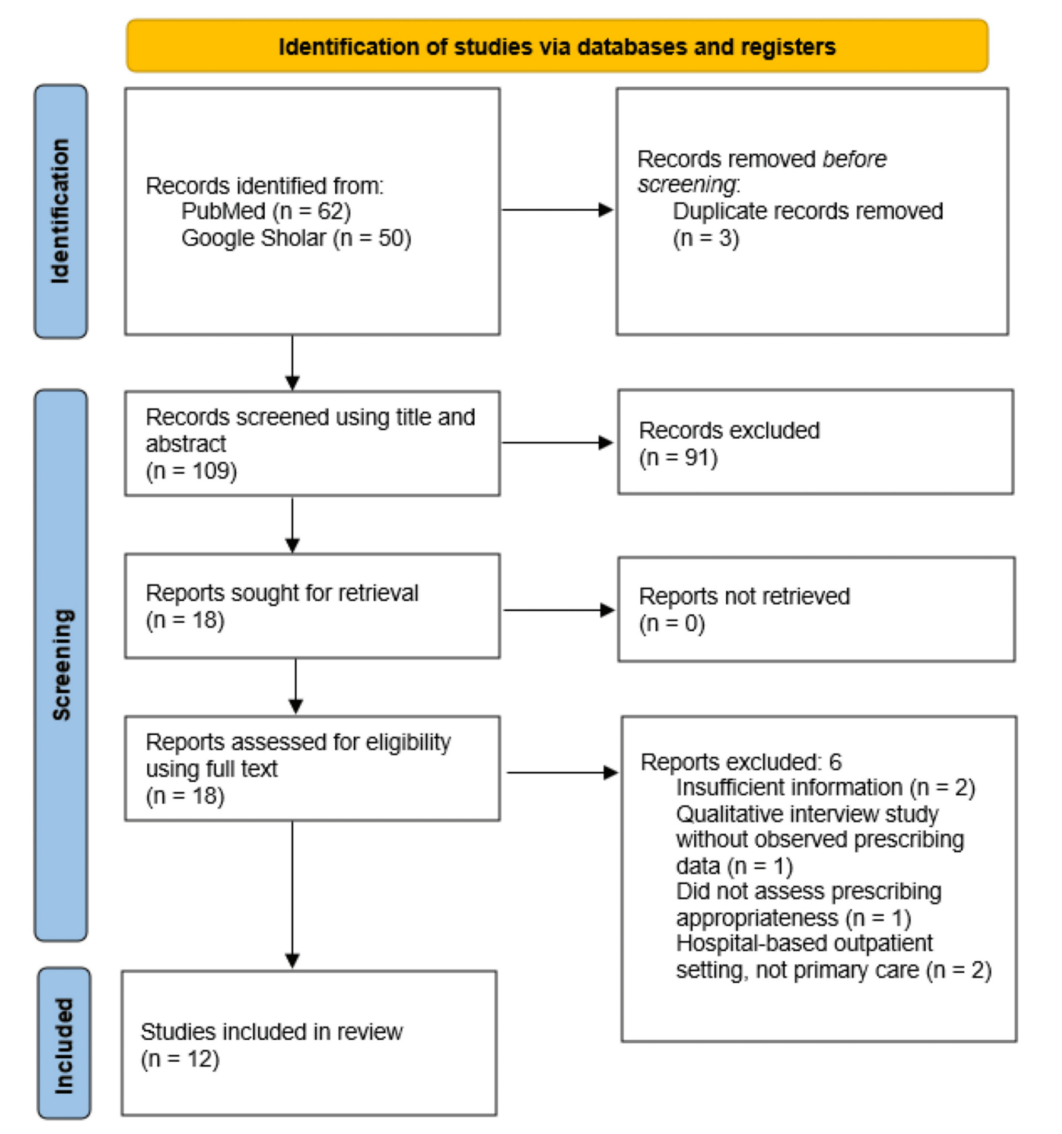
PRISMA flow diagram PRISMA: Preferred Reporting Items for Systematic Reviews and Meta-Analyses

Study selection was performed in two stages. First, using the title and abstract, followed by a full-text screening. The initial search yielded 112 results. After removal of duplicates, the remaining 109 results were screened using the title and abstract based on a predefined inclusion and exclusion criteria, resulting in the exclusion of 91 studies that did not meet the eligibility criteria, including studies conducted in non-primary care settings, studies with interventional designs, or those not reporting inappropriate antibiotic prescribing. Full texts of the 18 studies that met the inclusion criteria were obtained and assessed against the same criteria. Six studies were excluded, and the reasons for excluding studies at this stage were systematically recorded to ensure transparency and reproducibility of the selection process. Finally, 12 studies were included in this review. The flow diagram of the screening process is shown in Figure 1.

Data Synthesis

Due to the marked heterogeneity in terms of clinical characteristics, methodological approaches, and statistical analyses across the included studies - including study populations, definitions of inappropriate antibiotic prescribing, outcome measures, and analytical methods - a meta-analysis was not performed. Therefore, findings were synthesized narratively.

Results

Study Characteristics

A total of 12 observational studies conducted between 2021 and 2025 were included in this study, representing primary care settings across several countries, including the United States, France, the Netherlands, Italy, China, Pakistan, and Singapore [8-19]. The studies' populations encompassed adult and pediatric patients or both. All the studies were based in primary care facilities, including internal medicine, family medicine, pediatric, and general services. The definition of inappropriate antibiotic prescribing varied across the included studies, with definitions based on different criteria, such as national guidelines, prescription review standards, or study-specific classification systems. Key characteristics of the included studies are summarized in Table 1.

**Table 1:** Characteristics of the studies included in the qualitative synthesis URTI, upper respiratory tract infection; AURI, acute upper respiratory infection; ARS, acute rhinosinusitis; HIS, hospital information system; ICD-10, International Classification of Diseases, 10th Revision; AIR, antibiotic inappropriate rate; SPILF, Société de Pathologie Infectieuse de Langue Française (French Language Society for Infectious Diseases); ICPC, International Classification of Primary Care; UTI, urinary tract infection; MOH ACE, Ministry of Health Agency for Care Effectiveness in Singapore

Author (Year)	Country	Primary Care Setting	Population	Study Design	Definition of Inappropriate Antibiotic Use
Taxifulati et al. (2021) [13]	China	Primary healthcare institutions (community healthcare centers and stations)	Adult and pediatric patients (all ages)	Retrospective observational	Based on national Chinese prescription review standards and clinical treatment guidelines
Omer et al. (2021) [17]	Pakistan	Outpatient primary care departments of public health facilities	Adult and pediatric patients (all ages)	Cross-sectional observational outpatient prescription audit	Based on a tier-based classification, those with tier 3 diagnosis (conditions in which antibiotics are not indicated) were considered inappropriate
Zhao et al. (2022) [18]	China	Primary care institutions (urban and rural: community health service centers and stations, township hospitals, and village clinics)	Adults and pediatric patients (all ages)	Descriptive longitudinal observational database study using routinely collected data	Based on a tier-based classification, those with tier 3 diagnosis (conditions in which antibiotics are not indicated) were considered inappropriate
Chandra Deb et al. (2022) [15]	United States	Primary care clinics	Adults ≥18 years old attending for an URTI	Retrospective descriptive observational using electronic medical records	Unnecessary prescribing was defined as diagnosis of acute bronchitis, pharyngitis, nonspecific AURI, or ARS (where guideline-based criteria was not documented)
Wang et al. (2022) [16]	China	Primary care institutions (outpatient)	Children aged than 18 years old	Retrospective observational using the electronic HIS	Prescriptions were classified into incorrect spectrum of antibiotics (antibiotic choice does not match likely pathogens), unnecessary use (antibiotics prescribed for conditions not requiring antibiotics), and combined use of antibiotics (two or more systemic antibiotics prescribed for the same patient on the same day without indication) based on the Guiding Principle of Clinical Use of Antibiotics (2015, China) and USA Centers for Disease Control and Prevention guidelines.
Picca et al. (2023) [11]	Italy	Primary care pediatric clinics	Pediatric patients aged 0-5 years old	Prospective observational	The following diagnoses were classified as inappropriate indications for antibiotics: rhinitis, bronchitis, bronchiolitis, laryngitis, and non-streptococcal pharyngitis. these were then evaluated against national and international pediatric primary care guidelines
Fu et al. (2023) [12]	China	Primary healthcare facilities (outpatient)	Adult and pediatric patients (all ages)	Cross-sectional observational: prescriptions collected through the Chinese National Health Insurance system	The diagnosis was coded using ICD-10, then classified according to guideline-informed tiered diagnostic classification system into appropriate, potentially appropriate, inappropriate, or not linked to diagnosis
Neprash et al. (2023) [8]	United States	Primary care offices (internal medicine, family practice, and general practice specialties)	Adult patients (>15 years old)	Cross-sectional observational study, data collected from electronic health record systems	Prescription of an antibiotic for a primary diagnosis of upper respiratory tract infection
Li et al. (2023) [14]	China	Rural primary care institutions	Adult patients and children aged 0-5 years	Retrospective observational study using routinely collected primary care prescription data	Prescriptions involving an inappropriate spectrum of antibiotics, unnecessary antibiotic use, or both, based on national guideline recommendations, and quantified using the AIR
Charton et al. (2025) [9]	France	General practice clinics	Adult and pediatric patients (all ages)	Prospective observational study	According to SPILF guidelines
Sijbom et al. (2025) [10]	Netherlands	Primary care practices	Adult and pediatric patients (all ages)	Observational study - registry based on electronic medical record data that are linked with national socioeconomic registry	Based on Dutch primary care guidelines linking ICPC diagnostic codes to each prescription.
Ng et al. (2025) [19]	Singapore	National university polyclinics	Adult patients (>18 years old)	Retrospective observational	Prescriptions involving an inaccurate diagnosis or a diagnosis not in line with the 2023 Ministry of Health Singapore Clinical Guideline on UTI, or both. accuracy of the UTI diagnosis was evaluated against the MOH ACE clinical guidance on UTI

Quality Assessment of the Included Studies

The methodological quality of the included studies using the modified Newcastle-Ottawa Scale ranged from moderate to high. Most studies demonstrated clear definitions of inappropriate antibiotic prescribing and strong outcome assessment. Studies with limited sample size, single-center design, or descriptive analyses without adjustment for confounders had lower scores, while those using large electronic databases and multivariable analyses generally achieved higher scores. The detailed quality assessment scores of the included studies are presented in Table 2.

**Table 2:** Quality assessment of the included studies using the modified Newcastle-Ottawa Scale

Study	Selection (Maximum 5)	Comparability (Maximum 2)	Outcome (Maximum 3)	Total Score (Maximum 10)	Quality
Taxifulati et al. (2021) [13]	4	0	2	6	Moderate
Omer et al. (2021) [17]	3	0	3	6	Moderate
Zhao et al. (2022) [18]	4	2	3	9	High
Deb et al. (2022) [15]	4	2	3	9	High
Wang et al. (2022) [16]	4	2	2	8	High
Picca et al. (2023) [11]	4	2	2	8	High
Fu et al. (2023) [12]	4	0	3	7	Moderate
Neprash et al. (2023) [8]	4	2	2	8	High
Li et al. (2023) [14]	3	2	3	8	High
Charton et al. (2025) [9]	3	2	3	8	High
Sijbom et al. (2025) [10]	4	2	3	9	High
Ng et al. (2025) [19]	3	2	3	8	High

Prevalence of Inappropriate Antibiotic Prescribing

Although inappropriate antibiotic prescribing remained common in primary care across all the studies, the prevalence varied across different settings and populations. In a Chinese study that primarily focused on adult patients in an urban district, the prevalence was 10.9%. In contrast, in another study conducted in Southwest China and focused only on pediatric patients, a markedly higher prevalence of 80.5% was reported [13,16]. In the Netherlands, inappropriate antibiotic prescriptions accounted for 14.5% of all prescriptions, 24.7% in Italy, and 26% in France [9-11]. In the United States, 42.2%-55.7% of upper respiratory tract visits included inappropriate antibiotic prescriptions [8,15]. Meanwhile, in Singapore, the prevalence was 53.4% and 78% in Pakistani primary care [17,19].

Patterns of Inappropriate Antibiotic Prescribing

Among the studies that addressed patterns of inappropriate antibiotic prescribing by disease category, respiratory tract infections were dominant, and due to the high volume of consultations regarding them, they contributed to the largest share of inappropriate prescriptions even when their rate of inappropriate antibiotic prescribing was not the highest [10,13]. Among diseases of the respiratory system, bronchitis was consistently reported to be the diagnosis with the highest rate of inappropriate antibiotic prescribing, with a rate ranging from 73% to 100% [9,11,15,17,18]. This was followed by rhinitis and other diseases or symptoms, including rhinopharyngitis, fever, cough, non-infectious gastroenteritis, acute tonsillitis, urinary tract infection, common cold, and acute pharyngitis [9,11-13,17,18].

Two large retrospective primary care studies conducted in Southwest China reported high rates of inappropriate prescribing in several diagnostic categories. One of the studies examined patterns of prescribing among both adult and pediatric patients, while the other, which only focused on pediatric patients, observed a 100% rate of inappropriate antibiotic prescribing in unclassified symptoms, signs, and abnormal clinical/lab findings; an 88.6%-92.01% rate in skin and subcutaneous tissue diseases; a 31.89%-60.1% rate in diseases of the digestive system; and a 44.94%-60.4% rate in diseases of the genitourinary system [14,16].

Studies that examined antibiotic classes related to inappropriate prescribing reported that broad-spectrum antibiotic prescribing rates were very high. In contrast, narrow-spectrum antibiotic use was limited even when indicated, yet they accounted for a high inappropriate prescribing rate when not indicated [12,13,18,19]. Amoxicillin-clavulanate was consistently reported to be the most prescribed antibiotic and resulted in a high share of inappropriate antibiotic prescriptions [11,13,19]. Two studies that did not stratify antibiotic class by appropriateness stated that penicillins were the most frequently prescribed antibiotics [16,17]. One study highlighted the higher rate of inappropriate prescribing in children aged zero to five years old and patients of lower socioeconomic status and in rural areas [12].

Factors Associated With Inappropriate Antibiotic Prescribing

The factors associated with inappropriate antibiotic prescribing were identified at multiple levels, including patient, provider, and system levels. At the patient level, two studies concluded that the presence of fever independently increased inappropriate antibiotic use, particularly in respiratory tract infections [9,11]. Although some studies reported that age was not independently associated with inappropriate antibiotic prescribing, others consistently identified younger age as an associated factor. However, the definition of "younger age" varied across studies, with different age thresholds reported (e.g., < 1 year, < 2 years, 0-5 years, or < 6 years), limiting direct comparability [11,14,16,18]. Patient sex, however, was inconsistently reported as a factor associated with inappropriate antibiotic prescribing. Where effect sizes were available, the magnitude of association was small (e.g., OR 1.04 (95% CI: 1.03-1.05), OR 0.92 (95% CI: 0.90-0.95), OR 1.00). Notably, one study reported lower odds among males (OR 0.67 (95% CI: 0.65-0.68), with females as the reference), indicating higher odds among female patients, further highlighting inconsistency in findings [10,14,16,18].

At the clinician level, inappropriate prescribing was associated with male sex, older age, lower educational level, and greater clinical experience [14-16,18]. For male sex, where effect sizes were reported, the magnitude of association was generally small and inconsistent (e.g., adjusted OR 1.07 (95% CI: 1.05-1.08), OR 1.19 (95% CI: 0.94-1.52), adjusted OR 1.08 (95% CI: 1.04-1.11), and OR 1.00), suggesting limited clinical relevance.

System-level factors included rural practice settings, which were associated with higher inappropriate antibiotic prescribing [15,18]. Key findings of the included studies are summarized in Table 3.

**Table 3:** Key findings of the studies included in the qualitative synthesis Listed variables in the "factors" columns were independently associated with inappropriate antibiotic prescribing (↑ increased likelihood; ↓ decreased likelihood). Antibiotic classes reflect those reported in each study; not all studies evaluated class-specific inappropriate prescribing.

Author (Year)	Prevalence	Inappropriate indications	Antibiotic classes prescribed	Other patterns contributing to inappropriate prescribing	Patient factors	Physician factors	System factors
Taxifulati et al. (2021) [13]	10.9% of antibiotic prescriptions were inappropriate (derived from raw data provided by the author of the study)	Diagnosis resulting in inappropriate prescribing: acute bronchitis (17.6%); unspecified acute respiratory tract infection (14.4%); acute tonsillitis (9.9%); urinary tract infection (6.4%); common cold; acute pharyngitis	Second-generation cephalosporins, fluoroquinolones, and macrolides (89.3% of all prescriptions); β-lactam/β-lactamase inhibitors (amoxicillin-clavulanate) common; narrow-spectrum antibiotics underutilized	Adults (18–60 years); winter months, easy access to broad-spectrum antibiotics; limited diagnostic resources; weak guideline enforcement	Not reported	Not reported	Not reported
Omer et al. (2021) [17]	Out of all antibiotic-containing prescriptions, 78% were considered inappropriate	Rate of inappropriate prescriptions: bronchitis/bronchiolitis (100%), allergic rhinitis (75%); common cold/influenza (73%)	Penicillins (amoxicillin) (53%); cephalosporins (27%); fluoroquinolones (10.5%); macrolides (7.5%); tetracyclines (2%)	Not reported	Not reported	Not reported	Not reported
Zhao et al. (2022) [18]	Out of 1,287,678 antibiotic prescriptions, 653,335 (50.7%) were classified as inappropriate	Acute bronchitis; viral upper respiratory tract infections; influenza; non-specific respiratory symptoms	Broad-spectrum antibiotics (76.8%); WHO AWaRe classification: Watch antibiotics 54.9%. Access antibiotics 45.0%. Reserve antibiotics rare	Not reported	Younger age (<6 years)↑; male ↑	Male; Middle‑aged ↑; lower educational level ↑	Rural practice setting ↑; lower‑level primary care facilities ↑; out‑of‑pocket payment ↑
Chandra Deb et al. (2022) [15]	Unnecessary antibiotic prescription accounted for 42.2% of all prescriptions for URTI	Acute bronchitis (74.2%); acute rhinosinusitis without indication (45.8%); nonspecific acute upper respiratory infection (31.9%); pharyngitis without positive test (25.9%)	Not reported	Not reported	Male ↑; older age ↑; certain respiratory diagnoses (bronchitis and acute rhinosinusitis) ↑	More clinical experience ↑; high‑volume specialty practice such as urgent care ↑	Rural practice setting ↑
Wang et al. (2022) [16]	Overall inappropriate antibiotic use was 80.5%	Rate of inappropriate prescriptions: unclassified symptoms, signs, and abnormal clinical/lab findings (100%); diseases of the circulatory system (96.9%); skin and subcutaneous tissue diseases (88.6%); respiratory system diseases (81.9%); diseases of the digestive system (60.1%), diseases of the genitourinary system (60.4%); injury and poisoning (52.8%); diseases of the eye and adnexa (45.6%); certain infectious and parasitic diseases (30.0%); diseases of the ear and mastoid process (18.7%)	Penicillins (63.7%); cephalosporins (18.8%); lincosamides (5.8%)	Of all inappropriate prescriptions, 76.9% were due to unnecessary use, 2.4% due to incorrect spectrum,and 2.4% due to combined use; higher inappropriate prescribing in autumn-winter seasons	Younger age (0-1 year) ↑	Male ↑; older age ↑; lower professional titles (attending and resident physician roles) ↑; lower educational level ↑; certain ranges of longer clinical experience (6–10 years of service) ↑	Insurance coverage under the rural cooperative medical system ↑; later calendar quarters ↑
Picca et al. (2023) [11]	24.7% of all antibiotic prescriptions were inappropriate (derived from raw data provided by the author of the study)	Bronchitis (73%); rhinitis	Amoxicillin-clavulanate (41.6%); amoxicillin (38.4%); cephalosporins; macrolides	Not reported	Fever ↑; children <2 years ↑; children 3-5 years ↑ (bronchitis, pharyngitis)	Not reported	Not reported
Fu et al. (2023) [12]	70.5% of antibiotic prescriptions were classified as inappropriate	Upper respiratory tract infections, acute bronchitis, fever, cough, non-infectious gastroenteritis (tier-3 diagnoses; together 68.9% of inappropriate prescriptions)	Broad-spectrum antibiotics (82.2% of prescriptions; 70.1% inappropriate): second- and third-generation cephalosporins (most common among broad-spectrum); narrow-spectrum antibiotics (17.8% of prescriptions; 64.1% inappropriate)	Injectable antibiotic use (45.4%); multi-antibiotic prescribing (20.1%); children 0-5 years; rural residence; lower economic status	Not reported	Not reported	Not reported
Neprash et al. (2023) [8]	55.7% of upper respiratory tract infection visits involved an inappropriate antibiotic prescription	Not reported	Not reported	Not reported	Not reported	Shorter consultation time ↑	Not reported
Li et al. (2023) [14]	Overall inappropriate antibiotic prescribing rate was 66.19%	Rate of inappropriate prescriptions: musculoskeletal & connective tissue diseases (100%); symptoms/signs/R-codes (100%); skin & subcutaneous tissue diseases (92.01%); respiratory system diseases (69.57%); genitourinary system diseases (44.94%); digestive system diseases (31.89%)	Aminoglycosides (98.66% inappropriate); lincosamides (97.76% inappropriate)	Incorrect antibiotic spectrum (61.04%); unnecessary antibiotic use (5.15%); respiratory diseases were the highest contributor by volume; increased prescribing in winter months	Male ↑; younger age (0-5 years) ↑	Male ↑; older age (>35 years) ↑; more clinical experience (>11 years) ↑; lower educational level ↑; injectable antibiotic use ↑	Earlier years of visit (2017 vs 2018–2022)↑; out‑of‑pocket payment ↑
Charton et al. (2025) [9]	26% of all consultations involved inappropriate antibiotic prescriptions; among RTI consultations that resulted in antibiotic prescriptions, 60.5% were inappropriate	Bronchitis; rhinopharyngitis	Not reported	Not reported	presence of fever ↑; repeated consultations for the same episode of illness ↑	Diagnostic uncertainty ↑; incomplete clinical examination ↑; clear diagnostic explanation to patient ↓	Recent pharmaceutical representative visits ↑
Sijbom et al. (2025) [10]	14.5% of all antimicrobial prescriptions were inappropriate	Respiratory tract infections (39.6%); urinary tract infections	Not reported	Not reported	Female ↑; increasing age ↑; Turkish, Surinamese and Dutch‑Caribbean migration backgrounds ↑; single parent households ↑; presence of more comorbidities ↑	Not reported	Larger primary care practice size ↑; Friday prescriptions ↑
Ng et al. (2025) [19]	53.4% of all antibiotic prescriptions were inappropriate	Not reported	Most commonly prescribed: amoxicillin-clavulanate. Rate of inappropriate use: amoxicillin/clavulanate (42%); amoxicillin, cephalexin, ciprofloxacin, doxycycline, and trimethoprim-sulfamethoxazole (100%)	Non-guideline-concordant prescribing (69.0%); inaccurate diagnosis (17.8%); both non-guideline prescribing and inaccurate diagnosis (13.2%)	Drug allergies ↑	Increasing number of diagnoses per visit ↑	Clinic-level variation ↑

Discussion

Principal Findings

This systematic review was conducted to explore the inappropriate use of antibiotics in primary care settings. It included 12 recent observational studies (2021-2025) that were all conducted in primary care settings across multiple countries, representing a range of economic contexts, including high‑income, upper‑middle‑income, and lower‑middle‑income countries [8-19]. Although prevalence varied widely, inappropriate antibiotic use was found to be common across all settings, ranging from approximately 11% to 80%. This range reflected the high degree of variation between different countries, populations, and health systems. Notably, even within one country, that is, China, the prevalence varied widely [14,16]. This largely reflects the methodological heterogeneity across different studies rather than true epidemiological inconsistency. The study applying stricter definitions classified a broader range of prescriptions as inappropriate, including prescriptions with an incorrect spectrum of antibiotics, unnecessary use, and combined antibiotic use, resulting in substantially higher prevalence estimates. In contrast, the study used a more conservative definition, restricted inappropriateness to clear violations of antibiotic use, resulting in lower reported prevalence. Variation in study populations was another contributor; pediatric-focused research - where viral infections dominate, and there is a lower tolerance for empirical antibiotics - tends to report higher prevalence in comparison to research, including adult populations where greater diagnostic ambiguity is accepted and empirical coverage may be tolerated. Finally, geographical and system-level context may have influenced the findings, with the study conducted in Southwest China (a less resourced location) reporting a higher prevalence compared to the study conducted in Beijing, a more developed healthcare setting and stronger prescription policy enforcement. This heterogeneity should also be considered when interpreting associated factors, as differences in study populations, healthcare systems, and study designs may influence the observed relationships and limit direct comparability across studies.

Several included studies reported that respiratory tract infections were the primary clinical indication of inappropriate antibiotic prescribing. Multiple studies reported that acute bronchitis, viral upper respiratory infections, and nonspecific respiratory symptoms resulted in most unnecessary prescriptions. This consistency across diverse economic contexts illustrates the central role of respiratory tract infections in driving inappropriate antibiotic prescribing. In addition to respiratory tract infections, very high rates of inappropriate antibiotic prescribing were reported in non-specific clinical presentations. Two studies reported a 100% rate of inappropriate prescribing: symptoms/signs/R-codes in one study and unclassified symptoms, signs, and abnormal clinical or laboratory findings in another [14,16]. Such categories represent diseases where a confirmed infectious etiology was not found. When clinicians encounter such categories, they may prescribe antibiotics as precautionary measures in the absence of clear clinical indications. The consistency of these findings emphasizes that diagnostic uncertainty and non-specific coding practices may contribute to inappropriate antibiotic use in primary care.

Broad-spectrum antibiotics were consistently observed to be inappropriately prescribed, with amoxicillin-clavulanate repeatedly being the most prescribed agent. Inappropriate prescribing was influenced by several factors, including patient (particularly younger age and presence of fever), provider (male, low educational level, and high clinical experience), and system-level factors (rural settings). Overall, these findings indicate that inappropriate antibiotic prescribing is a multifactorial problem shaped by several factors rather than a single determinant.

The findings of this review are in line with existing literature on this topic. A recent scoping review stated that respiratory-related studies were among the most frequently represented clinical areas under inappropriate use of antibiotics and that the prevalence of inappropriate antibiotic use worldwide and across all income levels was found to be high [20]. These parallel the findings of the current review that respiratory tract infections, particularly bronchitis, accounted for most of the inappropriate antibiotic prescriptions, and that, across the several countries that the study included, prevalence was high regardless of income status. At a broader level, the World Health Organization highlighted that antimicrobial resistance affects countries of all income levels; this reinforces the findings of the current review and indicates that inappropriate antibiotic prescribing is a global challenge that needs to be addressed urgently [3].

Interpretation of Key Patterns and Factors

The predominance of respiratory tract infection diagnosis (particularly bronchitis) under inappropriate prescribing reflects the diagnostic uncertainty of these conditions. This is reinforced by the observed pattern of frequent inappropriate prescribing of broad-spectrum antibiotics, probably thought to be a safer option by the physician, when diagnostic certainty is low.

Under patient-level factors, physicians may associate young age (0-5 years) with more adverse outcomes. This, accompanied by parental pressure, may drive physicians to practice defensive prescribing. At the physician level, a more experienced practitioner was reported to have higher odds of inappropriate prescribing [14-16]. This may be due to inadequate exposure to recent antimicrobial stewardship programs as compared to younger, more recently educated practitioners. At the system level, particularly in rural areas, the higher workload, as well as the limited availability of diagnostic resources in comparison to urban areas, may limit the opportunity for proper clinical assessment and pressure practitioners to make rapid clinical decisions, resulting in inappropriate antibiotic prescribing.

Implications for Practice and Policy

Due to the interconnected causes of this issue, educational interventions alone are likely to be insufficient to effectively reduce inappropriate antibiotic prescribing. Thus, interventions at all three levels (patient, provider, and system) are needed. Patient education, specifically education focused on parents of young children, is known to improve antibiotic prescribing [21]. The findings of this review further support this, as several included studies reported that younger patient age was associated with inappropriate antibiotic prescribing [11,12,14,16,18]. Thus, educating parents of those patients may present a practical strategy to improve antibiotic prescribing.

As diagnostic uncertainty is associated with inappropriate antibiotic prescribing [9], addressing this issue is important to reduce it. Thus, strengthening diagnostic stewardship is an important aspect of improving antimicrobial stewardship. Point-of-care testing (POCT) tools provide rapid results that can guide clinicians in making accurate diagnoses; implementing them in primary care can improve antibiotic decision-making [22]. In addition to reducing the effects of unnecessary antibiotic use on patients, POCT can provide economic benefits by reducing healthcare costs [22].

Other implications include strengthening antimicrobial stewardship interventions at the primary care level by focusing specifically on self-limiting common diseases such as bronchitis and other respiratory tract infections, and by promoting the importance of adhering to guidelines when selecting antibiotics to treat them. This includes using narrow-spectrum antibiotics when needed. Delayed prescribing strategies can also be included to reduce antibiotic consumption [23]. In one observational study evaluating delayed antibiotic prescribing in primary care, 39.7% of patients did not collect the antibiotic prescription, while 64.3% obtained the antibiotic during the two-week follow-up period [23]. This shows that delayed prescribing reduced antibiotic use but did not eliminate it.

In light of the prescribing patterns observed in this review, continued medical education, regardless of practitioners' experience level, should be encouraged, with a specific focus on updated stewardship principles. Finally, improved access to diagnostic resources in rural areas needs to be addressed, which may reduce pressure on clinicians practicing in those areas. 

Strengths and Limitations

This systematic review has several strengths; it adhered to PRISMA 2020 methodology and applied strict inclusion criteria, focusing on studies that observed actual prescribing behaviors rather than theoretical assumptions. The findings clearly differentiated between descriptive patterns and regression-based factors.

However, some limitations should be taken into account. These include the heterogeneity in defining inappropriate antibiotic use. This heterogeneity may limit the comparability and interpretation of the reported prevalence, prescribing patterns, and associated factors. In addition, the included studies varied substantially in terms of healthcare systems and study populations, which may affect the generalizability of the findings across different settings.

Despite using predefined inclusion and exclusion criteria, the study selection process involved the exclusion of a large proportion of initially identified records, which may have introduced potential selection bias, and the use of Google Scholar, limited to the first 50 results, may have introduced selection bias and reduced reproducibility.

Furthermore, this study relied solely on observational studies. Thus, the identified associations do not imply causality and may be influenced by residual confounding. The study also used electronic health records to extract information. Although these records enable analysis of large populations by providing large-scale prescribing information, they may lack detailed clinical data, such as indications for antibiotic use, severity of illness, or presence of comorbidities, and may be subject to coding errors and inconsistencies.

Finally, effect sizes for associated factors were not consistently reported across studies, and where available, they were often small and variable, limiting assessment of their clinical significance.

Research Gaps and Future Directions

The studies included in this review did not present results from low-income countries. This is a significant gap in the literature. Access to diagnostics in these countries is often poor, which may heavily impact antibiotic prescribing. Moreover, the healthcare infrastructure and the regulation of antibiotic prescribing in these countries may differ. Another important gap is that all studies included were observational and focused on patterns and factors associated with inappropriate prescribing, and did not evaluate interventions to reduce inappropriate antibiotic prescribing in primary care. Thus, future research is needed to evaluate interventions aiming to reduce inappropriate antibiotic prescribing in primary care.

## Conclusions

This systematic review demonstrates that the prevalence of inappropriate antibiotic prescribing in primary care is high across diverse countries, with varying intensities across diverse economic contexts. Respiratory tract infections, particularly bronchitis, were the most frequent contributors to inappropriate antibiotic prescribing. In addition, broad-spectrum antibiotics were frequently inappropriately prescribed, with amoxicillin-clavulanate being the most common. Multiple factors influence inappropriate prescribing, including patient-level factors (specifically, patients of younger age), practitioner-level factors (particularly greater clinical experience), and system-level factors (rural practice settings). These findings highlight the multifactorial nature of inappropriate antibiotic prescribing in primary care and suggest that multifaceted strategies - such as strengthening stewardship programs in primary care, enhancing continuing medical education of practitioners, and developing clinical decision-support tools - may represent potential targets for intervention.

## References

[1] Otaigbe II, Elikwu CJ (2023). Drivers of inappropriate antibiotic use in low- and middle-income countries. JAC Antimicrob Resist.

[2] (2026). Antibiotic resistance threats in the United States. https://stacks.cdc.gov/view/cdc/82532.

[3] (2026). Antimicrobial resistance. https://www.who.int/news-room/fact-sheets/detail/antimicrobial-resistance.

[4] Murray CJ, Ikuta KS, Sharara F (2022). Global burden of bacterial antimicrobial resistance in 2019: a systematic analysis. Lancet.

[5] (2026). Inappropriate antibiotic prescribing leads to increased costs complications. https://www.pew.org/en/research-and-analysis/data-visualizations/2023/inappropriate-antibiotic-prescribing-leads-to-increased-costs-complications.

[6] Macy E (2020). Addressing the epidemic of antibiotic "allergy" over-diagnosis. Ann Allergy Asthma Immunol.

[7] Duffy E, Ritchie S, Metcalfe S, Van Bakel B, Thomas MG (2018). Antibacterials dispensed in the community comprise 85%-95% of total human antibacterial consumption. J Clin Pharm Ther.

[8] Neprash HT, Mulcahy JF, Cross DA, Gaugler JE, Golberstein E, Ganguli I (2023). Association of primary care visit length with potentially inappropriate prescribing. JAMA Health Forum.

[9] Charton L, Séverac F, Hansmann Y, Chambe J (2025). Factors influencing inappropriate antibiotic prescription in respiratory tract infections in general practice. Sci Rep.

[10] Sijbom M, Boelens M, de Boer MG, Numans ME (2025). Routine data registries as a basis to analyse and improve the quality of antimicrobial prescription in primary care. BMC Prim Care.

[11] Picca M, Carrozzo R, Milani GP (2023). Leading reasons for antibiotic prescriptions in pediatric respiratory infections: influence of fever in a primary care setting. Ital J Pediatr.

[12] Fu M, Gong Z, Zhu Y (2023). Inappropriate antibiotic prescribing in primary healthcare facilities in China: a nationwide survey, 2017-2019. Clin Microbiol Infect.

[13] Taxifulati Y, Wushouer H, Fu M (2021). Antibiotic use and irrational antibiotic prescriptions in 66 primary healthcare institutions in Beijing city, China, 2015-2018. BMC Health Serv Res.

[14] Li C, Cui Z, Wei D (2023). Trends and patterns of antibiotic prescriptions in primary care institutions in southwest China, 2017-2022. Infect Drug Resist.

[15] Chandra Deb L, McGrath BM, Schlosser L (2022). Antibiotic prescribing practices for upper respiratory tract infections among primary care providers: a descriptive study. Open Forum Infect Dis.

[16] Wang W, Yu S, Zhou X, Wang L, He X, Zhou H, Chang Y (2022). Antibiotic prescribing patterns at Children’s outpatient departments of primary care institutions in southwest China. BMC Prim Care.

[17] Omer S, Khan BT, Jalil O, Khan MW, Mehdi Q, Sharif M (2021). Inappropriate antibiotic use for respiratory infections in outpatient settings. J Fatima Jinnah Med Univ.

[18] Zhao H, Wang S, Meng R (2022). Appropriateness of antibiotic prescriptions in Chinese primary health care and the impact of the COVID-19 pandemic: a typically descriptive and longitudinal database study in Yinchuan city. Front Pharmacol.

[19] Ng LY, Goh LH, van der Lubbe SC, Koh SW (2025). Factors affecting antibiotic appropriateness in uncomplicated urinary tract infections in primary care. Sci Rep.

[20] Mulchandani R, Tiseo K, Nandi A, Klein E, Gandra S, Laxminarayan R, Van Boeckel T (2025). Global trends in inappropriate use of antibiotics, 2000-2021: scoping review and prevalence estimates. BMJ Public Health.

[21] McDonagh MS, Peterson K, Winthrop K, Cantor A, Lazur BH, Buckley DI (2018). Interventions to reduce inappropriate prescribing of antibiotics for acute respiratory tract infections: summary and update of a systematic review. J Int Med Res.

[22] Saha SK, Promite S, Botheras CL, Manias E, Mothobi N, Robinson S, Athan E (2023). Improving diagnostic antimicrobial stewardship in respiratory tract infections: a protocol for a scoping review investigating point-of-care testing programmes in community pharmacy. BMJ Open.

[23] Llor C, Moragas A, Cots JM (2022). Implementation of the delayed antibiotic prescribing strategy. Prospective observation study in primary care. Rev Esp Quimioter.

